# Improving Home Care Safety Among Informal Caregivers Through Immersive Digital Simulation: Secondary Analysis of 3 Coordinated Intervention Studies

**DOI:** 10.2196/85141

**Published:** 2026-07-09

**Authors:** José Joaquín Mira, Clara Pérez-Esteve, Eva Gil-Hernández, Almudena Arroyo-Rodríguez, Jesús María Aranaz-Andrés, Purificación Ballester, Irene Carrillo

**Affiliations:** 1 Departamento de Psicología de Salud, Universidad Miguel Hernández, Altamira Building, University Avenue, Elche, 03202, Spain, 34 966658984; 2 Grupo de Investigación ATENEA, Fundación para el Fomento de la Investigación Sanitaria y Biomédica de la Comunitat Valenciana, Alicante, Spain; 3 San Juan de Dios Foundation, San Juan de Dios School of Nursing and Physical Therapy, Comillas Pontifical University, Madrid, Spain; 4 Department of Preventive Medicine, Hospital Ramón y Cajal, Madrid, Spain; 5 Facultad de Farmacia y Nutrición, UCAM, Universidad Católica de Murcia, Murcia, Spain

**Keywords:** informal caregivers, home care, patient safety, psychoeducational training, virtual reality, augmented reality, cost-consequence analysis, caregiver burden

## Abstract

**Background:**

Informal caregivers perform complex home-care tasks but often lack structured training, causing preventable safety risks and burden. Technology-enhanced simulation provides practice; psychoeducational programs that flag risky activities may strengthen safety behaviors and self-efficacy. Comparing costs guides scalable caregiver training.

**Objective:**

This study compared the cost-consequences of traditional and immersive digital simulation for home care, focusing on costs, errors avoided, and caregiver-burden reduction.

**Methods:**

This is a prospective observational comparative cohort study with secondary cost-consequence analysis and incremental cost-effectiveness ratios (ICERs) versus control, expressed in €/error avoided and €/burden point reduced. Costs are reported in euros. A prespecified synthesis of 3 coordinated studies yielded 3 active arms plus a control cohort, implemented independently between August 2023 and July 2025 under a shared core protocol in comparable Spanish home-care settings. Arms were psychoeducation, virtual reality (VR), 360° video training (360VT), and standard education as control. Outcomes were 3-month changes in self-reported errors and caregiver burden, measured with the Zarit Burden Interview-7 (ZBI-7; 0–28). Costing adopted a societal perspective and included staff time, caregiver time, and development costs amortized over 3 years at 200 participants/year. Downstream health care use was modeled by applying 1%–5% of follow-up incidents (base case 2%) to a €1,257 composite unit cost. Combined costs equaled direct plus downstream costs, with no discounting over 3 months. Costs and consequences were reported in natural units, including incremental and pairwise comparisons.

**Results:**

A total of 282 caregivers were included (psychoeducational n=71, VR n=70, 360VT n=71, and control n=70). Incident changes over a 3-month period were: +0.44 (95% CI 0.06 to 0.82) for control, −0.51 (95% CI −0.89 to −0.13) for psychoeducational, −0.56 (95% CI −0.97 to −0.20) for VR, and −0.20 (95% CI −0.66 to 0.09) for 360VT. Combined cost/per participant (direct +2% downstream) was: €46.88 for control, €77.04 for psychoeducational, €105.60 for VR, and €42.97 for 360VT. ICERs versus control were: (1) for errors avoided, —€31.75/error (95% CI €19.78 to −€54.98) for psychoeducation, €58.72/error (95% CI €39.40 to −€97.89) for VR, and 360VT was dominant, saving €6.11 (95% CI €4.24 to −€10.63) per error avoided, with €3.91 (95% CI €3.52 to −€4.29) saved per participant; (2) for burden reduced (ZBI-7), −€55.85/point (95% CI €38.27 to −€98.68) for psychoeducational, €45.88/point (95% CI €32.12 to −€76.67) for VR, and 360VT was dominant, saving €6.41 (95% CI €4.42 to −€11.23) per point reduced, with €3.91 (95% CI €3.52 to −€4.29) saved per participant. Pairwise for burden were as follows: (1) VR versus psychoeducational, €38.59 (95% CI €26.89 to −€66.13) per extra point; (2) 360VT versus psychoeducational dominant; and (3) VR versus 360VT, €93.48 (95% CI €64.41 to −€163.85) per extra point.

**Conclusions:**

This real-world cost-consequence analysis innovatively compares immersive and nonimmersive training for informal caregivers from a societal perspective, using harmonized safety, burden, and downstream cost outcomes. Findings support stepped adoption: 360VT as a scalable default, VR for higher-risk tasks or caregivers with greater burden, and psychoeducation as a complementary low-cost strategy when resources are constrained.

## Introduction

### Challenge

The care of individuals with chronic illnesses or functional dependency is increasingly shifting from institutional settings to the home [1]. This transition is driven by both cost-containment policies promoting deinstitutionalization and by patients’ preference to remain in their familiar environments for as long as possible. This trend is unfolding in the context of population aging and rising demand for long-term care; in the European Union, the number of people in need of long-term care is projected to increase up to 38.1 million in 2050 [2]. At the same time, informal caregivers already provide a substantial share of support, with more than 1 in 8 adults aged 50 years and older reporting informal caregiving across Organisation for Economic Co-operation and Development countries, most of them being women [3]. In aging societies, such as Spain (where people aged 65 years and older represented 20% of the population in 2024), Italy (24.7% in 2025), and Japan (29.3% in 2024), family caregivers are assuming an increasing share of complex care at home [4-6]. However, this shift entails a growing burden on informal caregivers, who are often required to perform complex care tasks without having received structured or personalized training. This lack of technical and emotional preparation can compromise both patient safety and caregiver well-being [7-9].

### Simulation

Clinical simulation has become a cornerstone of health professions education, widely recognized for its effectiveness in improving technical skills, clinical reasoning, and transversal competencies, such as communication and teamwork [10-17]. Recent systematic reviews have documented substantial improvements in clinical performance, learner confidence, and decision-making in both medicine and nursing through the use of simulated environments [11,12,14]. These benefits, together with the opportunity to rehearse practice in settings that do not expose real patients to harm, have consolidated simulation as a safe and well-established pedagogical approach in regulated health training programs [15,16]. However, educational and economic effects vary by modality, implementation, and comparator, and greater fidelity does not necessarily translate into greater effectiveness [11,18]. Accordingly, simulation is best regarded as a safe and well-established pedagogical approach in regulated health training programs, with demonstrated educational benefits and economic potential whose value may differ according to setting and mode of delivery [16,18].

### Training Informal Caregivers

Despite its success in professional training, the use of simulation to train informal caregivers remains limited but is gaining momentum. Results have shown significant benefits of training based on simulation in mental health, perceived burden, and quality of life among informal caregivers, particularly when interventions combined educational, psychoeducational, and cognitive-behavioral strategies [19]. The most effective interventions typically integrate theoretical instruction, guided hands-on practice, and structured feedback, thereby enhancing comprehension, promoting skill retention, and facilitating the transfer of competencies to real-life home caregiving contexts [7,8]. Emerging evidence—mainly from pilot studies and quasi-experimental trials—has shown promising outcomes in contexts, such as dementia [20,21], cancer [9,22], and spinal cord injury [23]. Caregivers who participate in simulation-based programs report significant improvements in their self-efficacy and a greater sense of security in performing critical care tasks [24]. Moreover, they often perceive these programs as useful and feasible, further supporting their acceptability [25]. However, gaps remain in programs focusing on emotional and psychological support, and the need for more robust, transnational studies that comprehensively address the physical, emotional, social, and educational needs of informal caregivers throughout the care trajectory has been emphasized [19].

### Immersive Techniques

In recent years, immersive technologies have gained prominence as innovative training tools for home-based care. Interventions incorporating virtual reality (VR), augmented reality (AR), 360° immersive video guidance, and 3D scenarios and multisensory environments have shown potential in boosting caregiver motivation and engagement [26]. These approaches promote active learning through interactive and visually enriched experiences [20,27]. Several of these studies have explicitly addressed the concept of fidelity in simulation by incorporating technical, contextual, and emotional dimensions. While greater fidelity does not automatically imply greater effectiveness [28], considering these dimensions can improve learner immersion and may enhance the transfer of competencies in everyday caregiving situations.

In the past few years, immersive 360-degree video has emerged as a promising training tool in health care education [29]. By presenting real-world scenarios from a first-person perspective, it can enhance contextual realism, learner engagement, and situational understanding while remaining more accessible and easier to deploy than fully interactive immersive systems. This format is particularly relevant for caregiver education because it can be delivered through widely available devices, such as smartphones or tablets, requires less technical support than high-immersion systems, and allows repeated viewing at the user’s own pace. These features make it well-suited to home-care settings, where caregivers often face time constraints, limited opportunities to attend formal training, and a need for guidance that can be revisited during or immediately before everyday care tasks. In this sense, immersive 360° video may offer a practical balance between experiential richness and implementation feasibility, making it a promising option for caregiver education and just-in-time support in the home [30].

Extended reality (XR), encompassing VR, AR, and mixed reality, offers varying levels of immersion by extending sensory experience into digital environments [31,32]. As hardware and content costs have declined, interest has grown, and evidence generally indicates beneficial effects; however, findings are mixed, and head-to-head comparisons with low-tech simulators (eg, manikins) do not always show XR to be more cost-effective [33]. Moreover, XR typically entails resource-intensive content development and often requires simulation laboratories and trained support staff, which can increase implementation costs relative to lighter alternatives [34]. These uncertainties justify evaluating lighter, scalable alternatives (such as situated video guidance) alongside XR when designing caregiver training.

### Addressing Training Gaps in Home Care

Time constraints and competing responsibilities often make attendance at in-person courses unfeasible for informal caregivers [35]. Conceptual and empirical work on patient safety in the home identifies ill-prepared family caregivers as a key hazard domain, highlighting unmet training needs and the value of flexible, needs-based curricula delivered at the point of care [36].

Interest in technology-enhanced training approaches has therefore increased [37-39]; however, the adoption of immersive modalities in informal caregiver education remains limited, largely due to the perceived complexity and cost of developing immersive content and of the required infrastructure, such as simulation laboratories, specialized equipment and training materials, and trained personnel [40,41].

Although interest in immersive modalities for caregiver and health care training has grown, formal economic evidence remains limited and methodologically heterogeneous [33]. Systematic reviews of XR interventions in health care have identified relatively few full economic evaluations and substantial variation in costing methods, analytic perspectives, and outcome measures, which makes cross-study comparison difficult and limits decision-makers’ ability to judge whether the higher upfront costs of immersive technologies are justified compared with lighter or more traditional training formats [33,42]. In addition, the implementation literature shows that adoption depends not only on effectiveness but also on practical factors such as hardware requirements, content-development costs, workflow integration, and staff support [40,43,44]. Only a small number of studies have directly examined the costs of simulation modalities, and these have largely been conducted in professional training contexts rather than in informal caregiving [18,34]. In this context, a cost-consequence analysis (CCA) is particularly useful because it presents costs alongside multiple relevant outcomes in their natural units, allowing a pragmatic comparison of immersive and lower-intensity alternatives and informing efficient, scalable implementation in real-world home-care settings [45].

### Aim

To compare, from a societal perspective, the cost-consequence of traditional caregiver education versus immersive digital simulation-based training in home care, reporting disaggregated costs and outcomes (errors avoided, reduction in caregiver burden) to inform efficient, scalable adoption.

This study formed part of a broader program to strengthen safety in home care delivered by informal caregivers. It examined how distinct technology-enhanced training modalities prepare caregivers to perform common home-care tasks more safely and competently.

## Methods

### Study Design

This manuscript reports a secondary economic evaluation using a prospective observational comparative cohort design. The analysis was based on prospectively collected data from 3 coordinated studies that yielded 3 active intervention arms, together with a single contemporary control cohort used as a common comparator, all implemented under a shared core protocol in real-world health care settings in Spain between August 2023 and July 2025 [46-48]. Randomization was undertaken within the source studies, but not across training modalities. This economic evaluation is reported in accordance with the Consolidated Health Economic Evaluation Reporting Standards (CHEERS) 2022 statement [49].

Eligibility criteria, scenarios, and measurement procedures were prospectively harmonized to enable comparisons across modalities (VR, 360° video training [360VT], and psychoeducational program) against a suitable comparator (standard education or control). All groups were exposed to the same caregiving scenarios, adapted to reflect the typical tasks performed by participants in their home environments. A fourth group, receiving standard guidance, served as the control. Participants were recruited independently for each trial, and randomization was performed within each study. However, because the different training modalities were evaluated in separate trials, participants were not randomized across modalities. Therefore, comparisons across modalities should be interpreted as comparative rather than as evidence from a single randomized comparison.

A CCA [49] framework was applied from a societal perspective, incorporating both direct intervention costs and downstream health care costs, and presenting costs alongside multiple consequences in their natural units rather than aggregating benefits into a single monetary or utility metric. Consequences included the absolute reduction in self-reported caregiving incidents (errors avoided) and the reduction in caregiver burden measured with the 7-item Zarit scale (ZBI-7) [50]. Direct costs comprised professional time, caregiver time, and amortized development costs of educational materials, estimated under uniform assumptions across study arms. Results are reported disaggregated by outcome and intervention, with incremental comparisons versus the control cohort to inform interpretability within the CCA framework. The time horizon of the economic evaluation was 3 months, corresponding to the follow-up period of the study; therefore, no discounting was applied.

The primary effectiveness results of the individual trials have been reported elsewhere [46-48], each detailing outcomes for its respective modality. The protocol for all studies shared a common core—aligned scenarios, harmonized eligibility, and a unified measurement framework. This study reports a secondary comparative economic analysis integrating datasets from these parallel trials under a prespecified comparative framework to enable a comparative cost-consequence assessment of traditional versus immersive approaches. Using the shared core protocol, we harmonized variables, standardized resource use and costing assumptions, and applied a common analytic plan to estimate relative value across modalities.

### Setting

The study was conducted in Spain with the support of health care institutions located in Andalusia, Madrid, and the Valencian Community, including 6 hospitals, 2 primary care centers, and 2 health training schools. Baseline data were collected before the intervention, and follow-up assessments were conducted 3 months after completion of the training.

### Participants

Informal caregivers of individuals with chronic illnesses were recruited through primary care centers, community associations, and hospitals collaborating in this study. Health care professionals from these sites identified potential participants during the course of their routine activities, explained the study’s objectives and conditions, and invited eligible individuals to participate. Individuals who expressed interest were then formally invited to participate, provided they met the inclusion criteria.

Inclusion criteria were (1) age ≥18 years, (2) being the primary informal caregiver of an adult patient with chronic conditions requiring home care, (3) providing at least 15 hours of care per week, (4) no previous formal training in the use of virtual or AR technologies, and (5) provision of written informed consent. Exclusion criteria included (1) cognitive impairment or physical limitations preventing participation in the training activities, (2) lack of access to a device compatible with the training modalities (eg, smartphone or VR headset), and (3) inability or unwillingness to complete the full study procedures. Participants were recruited independently within each trial. As a result, allocation across training modalities was not randomized, and each dataset corresponds to participants enrolled in its respective study.

### Blinding

Participants could not be blinded to the training modality; however, within each study, participants were unaware of whether other training approaches were being used. Allocation concealment was ensured within trials. Researchers involved in allocation had no previous access to participant characteristics to avoid potential biases.

### Materials

To ensure clinical relevance and maximize the impact of the training, the development of educational materials for this study was guided by a needs-based approach, aiming to personalize the content according to the care recipient’s condition and the caregiver’s daily responsibilities. A list of priority caregiving situations was established by combining findings from previous studies on home care safety with the insights of 12 health care professionals, each with over 5 years of experience in home hospitalization or medium-stay care settings. Through semistructured interviews, these professionals identified the most common types of care they provided in home settings and proposed the educational content that should be included to ensure accurate training and effective caregiving performance. Their input helped shape the design of training materials across all delivery formats by highlighting the most frequent and preventable caregiving errors encountered in the home environment. As a result, a set of safety-critical caregiving tasks was defined and used as the foundation for all training content. These tasks were then organized into thematic blocks, each corresponding to typical caregiving challenges and routines. These blocks are presented in Multimedia Appendix 1.

### Psychoeducational Materials

A printed guide was developed outlining the learning objectives, structure, and contents of the sessions [51]. The content was informed by previous research on informal caregivers’ needs and focused on enhancing self-efficacy, health literacy, and safe caregiving practices. Educational materials included audiovisual resources and paper-based tools designed to support active learning and group discussion. Materials were developed by a multidisciplinary team trained to ensure consistency and alignment with caregivers’ everyday experiences, with a focus on patient safety related to medication management, mobility, hygiene, feeding, pressure injury prevention and skin care, and the use of medical devices, as well as on caregiver self-care to help limit the burden of care.

### Immersive Materials

The identified core contents were adapted across multiple simulation modalities, ensuring consistency in the caregiving scenarios while leveraging the specific advantages of each technological format. All immersive interventions—VR and 360VT—were built upon the same set of safety-critical caregiving tasks and thematic blocks.

The immersive training materials were developed in collaboration with a specialized simulation and immersive production team. A total of 15 daily caring scenarios were created. For the VR arm, fully interactive 3D scenarios were created, compatible with Meta Quest 2 devices (Reality Labs), allowing participants to engage in realistic caregiving activities within an immersive virtual environment. For the 360VT, immersive video format presented the same scenarios, enabling users to observe caregiving situations from a first-person perspective, supporting situated learning and contextual awareness.

In each format, the core scenarios remained aligned in content and structure, ensuring that participants in different arms of the study encountered comparable caregiving situations, with variations only in the degree of immersion, interactivity, and sensory engagement provided by the respective technologies.

### Standard Educational Materials

In addition to routine educational materials, control group participants were provided video demonstrations of the same caregiving activities used in the intervention arms, delivered in a passive format without interactive or immersive features.

### Simulation Fidelity

To some extent, all modalities addressed the key dimensions of simulation fidelity. Technical fidelity was highest in VR, which provided interactive 3D environments with task-specific manipulation and immediate feedback via a head-mounted display. By contrast, 360VT was a trigger-based experience without a registered digital overlay (no spatial tracking, anchoring, or occlusion); therefore, its 360VT technical fidelity did not involve true augmentation of the real scene, and interaction was limited to media controls and step prompts. Contextual fidelity was supported by designing scenarios that closely resembled actual home environments and caregiving conditions, based on input from experienced health care professionals. Emotional fidelity was incorporated by including situations with time-sensitive decisions, patient resistance, or emotional distress, aiming to replicate the stressors commonly encountered in real-life home care. However, the degree of emotional fidelity likely varied across formats, being most pronounced in immersive VR and 360VT and less so in passive formats such as standard videos.

### Interventions

Building on the standardized core content and materials described above, each study group underwent a distinct training approach tailored to a specific delivery format. While all participants were exposed to the same caregiving scenarios and thematic blocks, the structure, intensity, and learning dynamics of the interventions varied notably across groups.

Participants were recruited independently for each trial in different institutions. Consequently, allocation across training modalities was not randomized, and cross-modality comparisons should be interpreted as exploratory rather than causal.

In the control group, participants received standard educational materials that were commonly provided in public health care centers. These included printed guides and video recordings of typical home care routines. Materials were available for self-paced, individual viewing without interaction or supervision.

Participants in the control group continued to receive educational information in the same manner as typically provided by their health care centers. This usually included verbal explanations during clinical visits, printed informational materials, and occasional recommendations to consult external video resources. To standardize the control condition across sites, all participants in this group were additionally provided with a link to a curated set of educational videos developed specifically for this study. These videos presented appropriate and safe caregiving practices, tailored to the types of care tasks most commonly performed by each participant at home. Access to these materials was voluntary, allowing caregivers to use them at their own discretion and pace.

In the psychoeducational group, participants attended a structured program consisting of 2 participatory sessions, each lasting approximately 60-90 minutes and conducted in groups of up to 12 participants, with an interval of 1-2 weeks between sessions. The sessions combined theoretical instruction with practical exercises focused on common caregiving risks, stress management, and behavioral challenges. Guided group discussions allowed caregivers to reflect on their experiences and develop practical strategies to manage emotional burden and enhance patient safety at home. The structure, objectives, and core contents of the intervention are detailed in Multimedia Appendix 2.

In the VR group, participants trained using immersive VR headsets (Meta Quest 2) that allowed interaction with a selection of 3D caregiving scenarios simulating real-life tasks, such as safe medication administration, patient mobilization, and emergency response. The training emphasized technical, contextual, and emotional fidelity to replicate realistic home caregiving environments. From the full library of available scenarios, each participant was assigned 3‐4 modules tailored to the specific types of caregiving activities they regularly performed at home, ensuring a personalized and relevant training experience. The sessions were conducted in dedicated simulation laboratories set up within the collaborating centers and were supervised by personnel trained in the use of VR technologies. Participants were allowed sufficient time to repeat the scenarios and build competence, with specific attention paid to managing the learning curve associated with VR use to ensure a beneficial and engaging experience.

In 360VT, participants completed first-person immersive video scenarios of common home-care tasks using them as on-the-spot guidance while performing those tasks at home. Participants accessed immersive caregiving scenarios presented from a first-person perspective and ran on smartphones or tablets without registered digital overlays. The immersive format aims to enhance situational awareness during caregiving tasks by allowing caregivers to observe procedures from a first-person perspective. Each participant was assigned 3‐4 video modules tailored to their specific caregiving responsibilities, ensuring content relevance. Although participants could use the materials at their own pace, an initial group session was conducted to demonstrate proper use of the immersive materials and enhance the effectiveness of the learning experience. Participants were then free to revisit the educational materials independently at their own pace, reinforcing skill acquisition through repeated exposure. By embedding training into real-world caregiving contexts, this modality enabled situated learning and the immediate application of safe practices in the home at a reasonable cost, as suggested in previous studies.

All interventions were developed to maintain alignment in thematic content and caregiving challenges, enabling consistent comparison across modalities while exploring the added value of different simulation formats.

### Variables

There were 2 primary outcomes that were used to assess the cost-effectiveness of the interventions across the included trials. First, change in the number of self-reported caregiving incidents, including errors related to medication management, nutrition and food preparation, hygiene and bathing safety, mobility and transfers, wound care, and other routine caregiving tasks were calculated as the difference between incidents reported during the 3 months preceding the intervention and those reported for the 3-month follow-up period after the intervention; and second, change in caregiver burden, measured using the short version of the Zarit Burden Interview-7 item (ZBI-7) at baseline and at 3-month follow-up.

The main exposure variable was training modality, categorized into 4 groups—standard education (control), psychoeducational training, VR training, and 360VT.

Cost variables were estimated from a societal perspective and included staff time required to design and deliver the intervention, caregiver time investment to complete the training (limited to structured sessions), amortized development costs of educational materials, and modeled downstream health care usage associated with caregiving incidents using a composite unit cost derived from national health care estimates. All costs were expressed in euros (€), price year 2025. The average exchange rate during the study period, from August 2023 to July 2025, was approximately €1=US $1.087.

All data were collected using standardized procedures under a shared core protocol with harmonized eligibility criteria, caregiving scenarios, and measurement instruments across all intervention and control groups, ensuring comparability between cohorts.

### Data Sources and Measurement

Outcome data were collected using standardized questionnaires administered at baseline (preintervention) and at 3-month follow-up after completion of the intervention. Caregiving incidents were self-reported by participants, and caregiver burden was assessed using the ZBI-7. Cost data were estimated from a societal perspective based on standardized assumptions regarding staff time, caregiver time investment, and amortized development costs of training materials. Downstream health care usage associated with caregiving incidents was modeled using a composite unit cost derived from national health care cost estimates. The same measurement procedures were applied across all intervention and control groups. All datasets were collected under a shared core protocol with harmonized eligibility criteria, caregiving scenarios, and measurement procedures, ensuring comparability across intervention and control cohorts.

### Bias

Several measures were implemented to reduce potential sources of bias. All datasets were collected under a shared core protocol with prospectively harmonized eligibility criteria, caregiving scenarios, and outcome measurements to ensure comparability across intervention and control groups. Standardized questionnaires were used at baseline and at 3-month follow-up for all participants. Nevertheless, because allocation across training modalities was not randomized and caregiving incidents were self-reported, the possibility of residual selection and reporting bias cannot be excluded.

### Study Size

This comparative cost-consequence study is a secondary integration of parallel studies already published or under review (VR, immersive 360VT, and psychoeducational training) plus a contemporary control cohort, all built on a shared core protocol. This study used the achieved sample sizes of the original trials (approximately 70 participants per arm), which were powered a priori for their primary effectiveness outcomes. Those original calculations assumed approximately 70% baseline prevalence of medication errors, a 10-percentage-point absolute reduction, 2-sided α=.05, 80% power, and 15% attrition. For the present synthesis, all cases with complete cost and outcome data were included.

### Quantitative Variables

Quantitative variables were analyzed as continuous measures. Changes in caregiving incidents and caregiver burden were calculated as the difference between baseline and 3-month follow-up values. Cost variables were valued in 2025 euros and expressed per participant, derived from estimated staff time, caregiver time investment, amortized material costs, and modeled downstream health care usage. No categorization of quantitative variables was performed.

### Economic Evaluation

#### Overview

To assess the economic viability of the different training modalities, a direct cost analysis was conducted from a societal perspective. This was a theoretical estimation, including three components: (1) professional time, (2) caregiver time investment, and (3) amortized development costs of educational materials. The calculation assumes typical conditions and does not include other potential expenses such as transportation, accompanying the patient at home during the training, or any additional support needs that may arise in individual caregiving contexts. Insurance costs were not disaggregated, as they form part of institutional coverage. Interactions with other professionals (eg, social workers and physicians) that may influence caregiver outcomes were not considered. This section reports direct costs only; downstream health care costs are presented separately and added to direct costs in sensitivity analyses. All estimates are expressed in euros per participant (Table 1).

**Table 1.** Estimated direct cost per participant by study group in home care training for informal caregivers.

Intervention	Staff time (min)	Staff cost (€)	Caregiver time (min)	Caregiver cost (€)	Material cost (€)	Total direct cost per participant (€)
Control	15	5.34	75	10.56	3.33	19.23
Psychoeducational	120	42.68	120	22.53	3.33	68.54
Virtual reality	45	16.0	45	6.34	75.0	97.34
360VT^a^	15	5.34	45	6.34	15.0	26.68

a 360VT: 360° video training.

#### Professional Time

Staff time was monetized using the gross hourly cost for a registered nurse in Spain. The annual gross salary was estimated at €32,000; adding employer social security contributions (≈33%) yields €42,560 per year. Assuming a 37.5-hour workweek over 52 weeks, the hourly rate used was €21.34.

#### Caregiver Time (Consistency Rule)

To avoid bias, caregiver time was treated consistently across modalities; structured time required to complete the assigned module (eg, a 45‐75 min session) was included for all arms using the same rule (required minutes to complete the module), whereas optional, unstructured viewing was not valued in any arm. Caregiver time was valued at €8.45/hour (Spain’s 2025 minimum wage).

#### Educational Materials

Development costs were not fully allocated to this study but treated as reusable infrastructure. Accordingly, the educational materials budget comprised the development of training content (video, slides, VR, and tablet-based modules) amortized over 3 years with an assumed annual reach of 200 participants [34] (600 beneficiaries per format in total). Material costs covered design, production, iterative improvements of the training modules during testing, and adaptation for validation [52]. No additional infrastructure or equipment costs were included.

#### Intervention Delivery

Immersive interventions included staff-guided participation per caregiver (about 45 min for VR in simulation laboratories and about 15 min for 360VT in an initial group demonstration), after which 360VT materials were used independently at home. The psychoeducational intervention consisted of two 60-minute sessions facilitated by 2 professionals per group, requiring greater time investment by both caregivers and professionals.

#### Estimating Downstream Costs of Caregiving Errors

Given the high variability in outcomes and the lack of systematic follow-up regarding the physical or emotional consequences of caregiving errors for patients or caregivers in most home care settings, these downstream costs were estimated in a conservative manner. Research in Spain suggests that approximately 2% of self-reported caregiving errors result in events requiring emergency department attention [53]. To estimate the additional burden on the health care system, a base-case assumption was applied whereby 2% of self-reported home-care medication errors trigger downstream health care use. For each intervention group, 0.02 was applied to the number of follow-up incidents and multiplied by a composite unit cost of €1257.03 per incident (emergency department visit €127.21+family physician visit €68+standard blood panel including phlebotomy €85+one inpatient day €976.82). Downstream costs were added to direct costs (societal perspective). In sensitivity analyses, this proportion was varied from 1% to 5% while holding unit costs constant (Table 2).

Table 3 presents the combined cost per participant under the societal perspective, obtained by adding downstream health care use to direct costs. Under the base case, the combined cost per participant was €46.88 for the control group, €77.04 for the psychoeducational intervention, €105.60 for the VR intervention, and €42.97 for the situated immersive video guidance intervention (Table 3).

**Table 2.** Projected downstream health care costs by study group in home-care caregiver training. Estimates are based on a composite unit cost (€1257.03) including emergency care, primary care, laboratory tests, and hospitalization, assuming a base-case incidence of 2% and a sensitivity range of 1%‐5%.

Group	Total downstream health care cost (€)	Cost per participant (€)	Total sensitivity 1%‐5% (€)	Sensitivity 1%‐5% – per participant (€)
Control (n=70)	1935.83	27.65	967.91-4839.57	13.83-69.14
Psychoeducational (n=71)	603.37	8.50	301.69-1508.44	4.25-21.25
Virtual reality (n=70)	578.23	8.26	289.12-1445.58	4.13-20.65
360VT^a^ (n=71)	1156.47	16.29	578.23-2891.17	8.14-40.72

a 360VT: 360° video training.

**Table 3.** Combined cost per participant by study group from a societal perspective, including direct intervention costs (staff time, caregiver time, and materials) and projected downstream health care costs (base case: 2%).

Group	Direct cost per participant (€)	Downstream cost per participant at 2% (€)	Combined cost per participant (€)
Control (n=70)	19.23	27.65	46.88
Psychoeducational (n=71)	68.54	8.50	77.04
Virtual reality (n=70)	97.34	8.26	105.60
360VT^a^ (n=71)	26.68	16.29	42.97

a 360VT: 360° video training.

### Statistical Analysis

The primary analytic framework was a comparative cost-consequence assessment presenting disaggregated costs alongside multiple consequences in natural units. As complementary efficiency metrics, incremental comparisons versus control were summarized using the incremental cost-effectiveness ratio (ICER), calculated as the difference in costs divided by the difference in effects between interventions (€/error avoided; €/ZBI-7 point reduced). Dominance was declared when an option was less costly and more effective. Pairwise ICERs between active interventions were reported for context. Deterministic sensitivity analyses varied the downstream-use proportion (1%‐5%), holding unit costs constant.

### Cost-Effectiveness Analysis

The ICER was calculated separately for each outcome of interest, reflecting the cost per unit of improvement in (1) self-reported caregiving errors and (2) caregiver emotional burden, using the following formula:


$$ICER_{A,B}=\frac{Cost_{A}-Cost_{B}}{Effect_{A}-Effect_{B}}$$

where “Cost” includes direct costs per participant (staff time, caregiver time, and amortized material cost) and “Effect” is the observed change in a specific outcome between the intervention and comparator groups.

Each ICER reflects the incremental cost per additional unit of improvement in a single dimension of caregiver performance or well-being.

Comparisons were conducted in 2 phases. First, each intervention was compared with the control group to determine the absolute cost-effectiveness of the training modality. Second, pairwise comparisons between interventions were made to explore relative efficiency across delivery formats. No subgroup or heterogeneity analyses were conducted, as the study was not powered for stratified comparisons across participant characteristics. This approach provides a comprehensive assessment of which modality delivers the greatest improvement per euro spent, both in absolute terms (vs control) and in relative terms (vs other active formats).

Effects were defined as positive improvements (eg, absolute reduction in incident rate, decrease in Zarit mean score). ICERs are reported as cost per unit improvement; dominance is declared when an option is less costly and more effective.

Finally, a deterministic sensitivity analysis was conducted to assess the robustness of the results. The proportion of reported incidents assumed to trigger downstream health care use was varied from 1% to 5% (base case 2%). For each proportion, downstream costs were recomputed and added to direct costs under the societal perspective, and ICERs were recalculated for both outcomes (errors avoided and reduction in caregiver burden), with the resulting ranges reported.

Uncertainty around incremental costs and effects was further explored using nonparametric bootstrap resampling (5000 replications). For each replication, incremental cost and effect estimates were calculated and plotted on cost-effectiveness planes, allowing visualization of the joint distribution of incremental costs and outcomes for each intervention relative to the control group.

No missing data were present in the final analytical dataset.

### Ethical Considerations

Ethical approval for this study was prospectively obtained from the Ethics Committee of Sant Joan d’Alacant University Hospital (December 22, 2021, project 21/063; February 1, 2023, project 22/79; and February 1, 2023, project 22/80). Before enrollment, all participants received detailed information about each study and provided written informed consent. Data collection procedures conformed to current Spanish legislation on research involving human participants and followed the ethical principles outlined in the Declaration of Helsinki (October 2024 revision). All study data were analyzed in anonymized form, and personal identifiers were removed before analysis to ensure participant privacy and confidentiality. No images included in the manuscript or supplementary materials permit the identification of individual participants or users. Participants did not receive financial compensation for their participation in the study.

## Results

### Overview

Data from a total of 282 informal caregivers were included across coordinated studies evaluating 3 training modalities+control group: 71 in the 2-session psychoeducational program, 70 in VR training, 71 in immersive 360VT, and 70 in the control group. Most participants were women (219/282, 77.7%), with a mean age of 40.5 (SD 18.8) years (Table 4).

**Table 4.** Sample characteristics of informal caregivers and care recipients by study group in home-care caregiver training.

Characteristics	Control	Psychoeducational	Virtual reality	360° video training
Participant				
Male, n (%)	18 (25.7)	15 (21.1)	12 (17.1)	17 (23.9)
Female, n (%)	51 (72.6)	56 (78.9)	58 (82.9)	54 (76.1)
Nonbinary, n (%)	1 (1.4)	0 (0)	0 (0)	0 (0)
Age (y), mean (SD)	55.8 (11.6)	50.9 (12.8)	33.4 (20.2)	23.4 (5.7)
Caregiving				
Relative, n (%)	66 (94.3)	31 (43.7)	49 (70)	51 (71.8)
Living together, n (%)	52 (74.3)	33 (46.5)	27 (38.6)	20 (28.2)
Hours per day, mean (SD)	13.3 (8.8)	10.2 (9.9)	10.3 (9.5)	8.8 (9.3)
Patient				
Sex (females), n (%)	35 (50)	18 (25.4)	35 (50)	43 (60.6)
Age (y), mean (SD)	75.6 (14.1)	76.5 (18.8)	66.9 (27.7)	75.4 (19.8)
Barthel (<60 points), n (%)	41 (58.6)	43 (60.6)	41 (58.6)	41 (57.7)
Medications taken daily, mean (SD)	7.7 (4.0)	4.7 (3.0)	4.6 (4.4)	4.4 (3.4)
Uses medication dispensing devices, n (%)	41 (58.6)	29 (40.8)	32 (45.7)	37 (52.1)
Degree of recognized dependency, n (%)	23 (32.9)	43 (60.6)	35 (50)	48 (67.6)

### Self-Reported Caregiving Errors

Training across modalities was contributing to reducing the number of safety incidents at home (Table 5). Over 3 months, self-reported incidents per participant changed by +0.44 in the control group, −0.51 in the psychoeducational group, −0.56 in the VR group, and −0.20 in the 360VT group (Table 5). Relative to control, the corresponding net reductions were 0.95 (psychoeducational), 1.00 (VR), and 0.64 (360VT).

**Table 5.** Cost-effectiveness analysis of caregiver training modalities in home-care settings, including incremental costs, effects, and ICERs^a^.

Measure and group	Control (n=70)	Psychoeducational (n=71)	Virtual reality (n=70)	360VT^b^ (n=71)
Cost per participant (€)^c^	46.88	77.04	105.60	42.97
∆ Cost (€) using the control group as comparator	—^d^	30.16	58.72	−3.91
∆ Cost (€) using the psychoeducational group as comparator	—	—	28.56	−34.07 (dominant lower cost; ≈€486.7 saved per point; €34.07 saved per participant)
∆ Cost (€) using the virtual reality group as comparator	—	—	—	−62.63
Self-reported errors made by participants				
Pre	0.66	0.85	0.89	0.85
Follow-up	1.10	0.34	0.33	0.65
Absolute change	0.44	−0.51	−0.56	−0.20
∆ Effectiveness versus control group	—	0.95	1.00	0.64
∆ Effectiveness versus psychoeducational group	—	—	0.05	−0.31
∆ Effectiveness versus virtual reality group	—	—	—	−0.36
ICER (€/unit improvement using the control group as comparator)	—	31.75 (95% CI 19.78‐54.98)	58.72 (95% CI 39.40‐97.89)	Dominant (≈€6.11, 95% CI €4.24-€10.63 saved per error; €3.91, 95% CI €3.52-€4.29 saved per participant)
ICER (€/unit improvement using the psychoeducational group as comparator)	—	—	571.20 (95% CI −2692.50 to 2794.73)	109.90 (95% CI 58.85‐366.42)
ICER (€/unit improvement using the virtual reality group as comparator)	—	—	—	173.97 (95% CI 101.08‐497.8)
Emotional burden experienced by participants				
Pre	9.10	7.58	8.14	9.01
Follow-up	8.87	6.81	6.63	8.17
Absolute change	−0.23	−0.77	−1.51	−0.84
∆ Effectiveness versus control group	—	0.54	1.28	0.61
∆ Effectiveness versus psychoeducational group	—	—	0.74	0.07
∆ Effectiveness versus virtual reality group	—	—	—	0.67
ICER (€/unit improvement using the control group as comparator)	—	55.85 (95% CI 38.27‐98.68)	45.88 (95% CI 32.12‐76.67)	Dominant (≈€6.41, 95% CI €4.42–€11.23 saved per point; €3.91, 95% CI €3.52–€4.29 saved per participant)
ICER (€/unit improvement using the psychoeducational group as comparator)	—	—	38.59 (95% CI 26.89‐66.13)	Dominant (cost saving €34.07)
ICER (€/unit improvement using the virtual reality group as comparator)	—	—	—	93.48 (95% CI 64.41‐163.85)

a ICER: incremental cost-effectiveness ratio.

b 360VT: 360° video training.

c Including downstream costs of caregiving errors.

d Not applicable.

### Emotional Burden

Mean burden scores declined from 9.10 (SD 6.14) to 8.87 (SD 6.07) in controls (Δ=−0.23, SD 6.25), 7.58 (SD 6.48) to 6.81 (SD 4.83) in the psychoeducational group (Δ=−0.77, SD 5.73), 8.14 (SD 3.99) to 6.63 (SD 5.52) in the VR group (Δ=−1.51, SD 5.36), and 9.01 (SD 5.13) to 8.17 (SD 4.56) in the 360VT group (Δ=−0.84, SD 5.69; Table 5). Relative to control, the additional reductions were 0.54 (psychoeducational), 1.28 (VR), and 0.61 (360VT) points per participant.

### Cost-Effectiveness Analysis

Table 5 summarizes costs, effectiveness outcomes, and ICERs for each intervention compared with the control group. The incremental cost per error avoided was €31.75 (95% CI €19.78-€54.98) for the psychoeducational program, €58.72 (95% CI €39.40-€97.89) for VR, and dominant for 360VT (saving approximately €6.11 per error avoided; €3.91 saved per participant).

For caregiver burden (ZBI-7 total), ICERs versus control were €55.85 (95% CI €38.27-€98.68) for the psychoeducational intervention, €45.88 (95% CI €32.12-€76.67) for the VR intervention, and dominant for 360VT (saving approximately €6.41 per point reduced; €3.91 saved per participant). Pairwise, VR versus psychoeducational cost per additional ZBI-7 point reduced (€38.59, 95% CI €26.89-€66.13); 360VT versus psychoeducational was dominant (lower cost, slightly greater reduction); and VR versus 360VT cost per additional point reduced (€93.48, 95% CI €64.41-€163.85).

The CIs indicate moderate uncertainty around several ICER estimates, particularly for comparisons involving psychoeducational and VR interventions.

### Primary Framework: CCA

**Figure 1.**
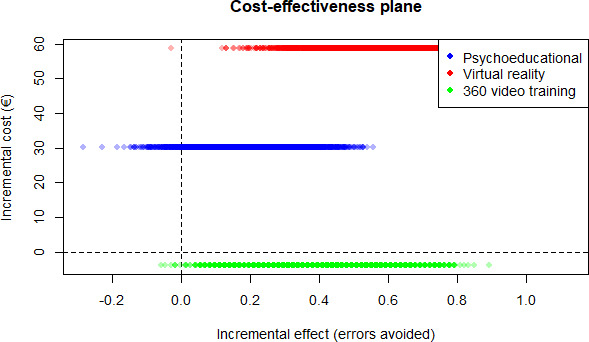
Cost-effectiveness plane for caregiving errors.

ICERs are reported as complementary incremental metrics versus control; dominance indicates lower cost and greater effect. To explore the uncertainty around incremental costs and effects, cost-effectiveness planes based on bootstrap resampling were generated. For the caregiving error outcome (Figure 1), psychoeducational and VR interventions were associated with higher costs and greater effectiveness compared with the control group (north-east quadrant), whereas the 360VT was both less costly and more effective (south-east quadrant), indicating dominance.

**Figure 2.**
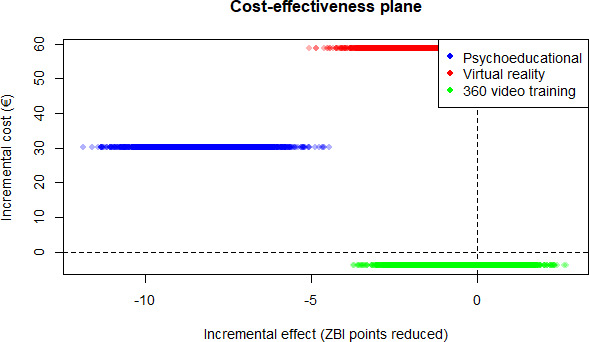
Cost-effectiveness plane for caregiver burden (ZBI-7). ZBI: 7-item Zarit scale.

A similar pattern was observed for emotional burden (Figure 2), where the 360VT remained dominant while psychoeducational and VR training showed higher effectiveness at higher costs.

### Sensitivity Analysis

Deterministic sensitivity analyses varied the proportion of follow-up incidents assumed to trigger downstream health care use from 1% to 5% (base 2%), holding unit costs constant (composite €1257.03 per incident: ED €127.21+family physician €68+standard blood panel €85+one inpatient day €976.82). Using the updated cost per participant figures (control €46.88, psychoeducational €77.04, VR €105.60, and 360VT €42.97), base-case ICERs versus control are €31.75 per error avoided for the psychoeducational program, €58.72 for VR, and dominant for 360VT (≈€6.11 saved per error avoided, €3.91 per participant). For caregiver burden (ZBI-7 total), base-case ICERs versus control are €55.85 per point for the psychoeducational program, €45.88 for VR, and dominant for 360VT (≈€6.41 saved per point; €3.91 per participant). Across 1%‐5% downstream-use scenarios, ICERs versus control improve (decrease) as the assumed downstream share rises, with 360VT becoming increasingly cost-saving; VR consistently yields the largest absolute effects on both outcomes, while the psychoeducational option remains the lowest cost per error avoided among nondominant alternatives.

## Discussion

### Findings

This comparative, real-world CCA indicates that all active modalities improved safety outcomes relative to standard education. While our base economic horizon was 3 months, higher upfront costs associated with simulation-based options are primarily absorbed over multiyear reuse through fixed-cost amortization; the principal constraints are logistical (eg, space for VR laboratories or group sessions) and staffing [43]. When projected over a multiyear horizon (≈3 y), even the most capital-intensive option—VR—becomes highly efficient compared with traditional information-delivery approaches, owing to amortization of development and set-up costs. However, the intervention groups were recruited in 3 independent trials; therefore, allocation across modalities was not randomized, and cross-modality comparisons should be interpreted as exploratory.

Findings suggest that 360VT delivered meaningful gains at a very low per-participant cost and was cost-saving versus control, making it the most economically efficient option for broad deployment. VR achieved the largest absolute improvements, yielding the greatest reductions in both self-reported errors and caregiver burden at a modest incremental unit cost. Psychoeducational sessions were cost-efficient for errors avoided but less efficient for burden reduction than 360VT and VR. Sensitivity analyses varying downstream usage (1%‐5%) did not alter the qualitative conclusions; 360VT became increasingly cost-saving as assumed downstream use rose.

Adoption studies of AR in complex clinical settings report good acceptability and perceived utility but also pragmatic barriers (device, workflow, and support), aligning with our feasibility and logistics observations for home care [44]. In parallel, positive economic findings for VR in mental health (eg, the gameChange trial) reinforce that with scale and content reuse, VR can deliver economic value across clinical domains [54].

Taken together, the findings support a pragmatic, stepped implementation strategy: deploy 360VT as the scalable default (improvement with cost savings), add VR selectively for higher-risk tasks or for caregivers with elevated burden (largest incremental effects at modest marginal cost), and use psychoeducation to extend low-cost error reduction where resources for immersive content development or in-person training time are constrained. In practical terms, 360VT should anchor routine competencies—mobility and transfers (bed-to-chair, repositioning schedules, and safe use of aids), feeding and hydration (positioning and aspiration precautions), basic wound or skin care and pressure-injury prevention (daily inspection and offloading), everyday hygiene, and nondevice medication routines (pillbox setup, timing, and double-checks)—by providing first-person, just-in-time prompts during real task execution at home [52]. VR should be reserved for procedures with low tolerance for error or high cognitive load—pen-device medication administration (dose dialing, priming, site rotation, and sharps disposal), home medical devices (nebulizers, enteral feeding pumps, oxygen therapy, and continuous positive airway pressure and bilevel positive airway pressure interfaces), aseptic or clean techniques (drug reconstitution and sterile handling), critical hand hygiene moments, and time-pressured emergencies (choking, hypoglycemia, and injurious falls)—where immersive rehearsal and immediate feedback yield the greatest incremental benefit. Based on the findings of this study and consistent with recent evidence, VR may be particularly useful for procedures with low tolerance for error or high cognitive load, especially those requiring stepwise execution, situational awareness, and immediate feedback [31]. This may include medication-device handling, home medical devices, aseptic or clean techniques, critical hand hygiene moments, and time-pressured emergencies. Psychoeducational sessions should run in parallel as a low-cost complement to strengthen hazard recognition and safety planning, with emphasis on medication risk identification (high-alert drugs, polypharmacy flags, and look-alike or sound-alike issues), red-flag escalation, checklist use, and coping strategies for burden, especially in settings where immersive content cannot yet be produced or deployed at scale [55].

### Innovations

This study positions informal caregivers as an integral part of home-based care, recognizing that the support they provide often constitutes a continuation of care previously delivered in hospital or ambulatory settings [56]. As health and social care systems progressively transfer care responsibilities to the home, patient safety should be considered a central objective of this transition. By framing caregiver training and its economic evaluation within this broader transformation, the study contributes a clinically meaningful perspective that is in line with recent efforts to better understand and address the role of informal caregivers in the safety of care recipients [1].

This study is innovative in providing a real-world comparative CCA of multiple technology-enhanced training modalities for informal caregivers under a shared protocol and from a societal perspective. In a field where previous studies have largely focused on feasibility, acceptability, or single-modality evaluations, and where formal economic evidence remains limited and methodologically heterogeneous [25,33,42], this study compares immersive and nonimmersive approaches through an explicit economic framework that integrates harmonized safety outcomes, caregiver-burden outcomes, and downstream costs. In doing so, it adds implementation-oriented comparative economic evidence to a field where cost analyses remain scarce and offers a more decision-oriented basis for selecting training strategies in home-care services [42].

### Findings in the Context of Previous Literature

These results are broadly consistent with previous work positioning immersive technologies as potentially cost-effective once development and hardware costs are amortized and user numbers scale [33,57]. In our setting, the main obstacle to implementing VR is the upfront investment; however, our VR unit costs and amortization assumptions align with ranges reported elsewhere, supporting the plausibility of our estimates and the medium-term value proposition of VR [25]. At the same time, VR may face practical constraints in home-care contexts—dedicated spaces, staff time, and access to equipment—constraints that are less prominent in studies with professionals or students who can attend on-site training [43]. By contrast, 360VT can be delivered on tablets or smartphones at home during routine care, avoiding travel and scheduling barriers [29,30]. Psychoeducational sessions, while valuable, retain fixed in-person costs even when materials are reusable [19,55].

In our cohort, the per-participant direct cost for VR was around €105, which is practically equivalent—in nominal terms—to the estimate by Farra et al [34] in the United States (US $115.43 per participant). While settings and cost components differ, this proximity suggests comparable orders of magnitude across contexts.

Quantitatively, technology-enhanced training delivered meaningful safety gains at low unit cost, supporting a stepped approach. 360VT was dominant versus control (lower cost and greater effect), with an estimated saving of about €6.11 per error avoided and €6.41 per ZBI-7 point (and €3.91 saved per participant). VR produced the largest absolute effects at modest incremental cost (€58.72 per error avoided; €45.88 per ZBI-7 point). The psychoeducational program was efficient for error reduction (€31.75 per error avoided) but less efficient than VR for burden reduction (€55.85 per ZBI-7 point). Taken together, these results support a 3-tier implementation—deploy 360VT as the scalable default, add VR selectively for higher-risk tasks or caregivers with elevated burden, and use psychoeducation to extend low-cost error reduction when resources or time for in-person training are limited.

In practice, this potentially higher appeal of VR may support adherence to selective modules [58]; nevertheless, this should be verified specifically in informal caregivers, rather than extrapolated from student populations. These observations echo reports that virtual modalities can achieve more favorable cost-utility than traditional simulation under realistic reuse and scale assumptions (eg, when materials are used across successive cohorts) [18,34]. Nevertheless, most published studies emphasize usability, acceptability, and feasibility rather than full economic evaluation; a smaller subset focuses on cost-minimization from a payer perspective or on outcomes such as anxiety in patients or training effects in professionals [25]. Standardized costing measures remain scarce, which limits cross-study comparability and underscores the need for common reporting frameworks in future research [42].

Taken together, these findings translate into a pragmatic stepped pathway for implementation, deploy 360VT as the scalable default for routine, sequence-sensitive tasks (mobility or transfers, feeding or hydration, basic wound or skin care or pressure-injury prevention, everyday hygiene, and nondevice medication routines). Use VR selectively for high-risk or high-cognitive-load procedures (pen-device medication administration, home devices, aseptic or clean techniques, critical hand-hygiene moments, and emergencies). Add psychoeducation to enhance medication-risk recognition, safety planning, and coping where immersive content or in-person time is constrained.

### What This Study Brings to the Field

This study helps make the safety problem in home care more visible by highlighting the frequency of self-reported caregiving errors and relating them to caregiver burden and intervention costs. In doing so, it draws attention to the often-overlooked safety challenges faced in households and to the additional strain that these situations place on informal caregivers [59,60]. The findings further indicate that the cost of achieving improvements in caregiver burden while reducing errors may be relatively low, reinforcing the societal relevance of investing in this area. By making the costs of different training modalities explicit, the study provides information that may support decision-making at different levels, including service organizations, managers, and health planners. It also strengthens the rationale for caregiver training programs as a safety strategy [39] and shows that approaches such as VR and immersive video, although associated with higher upfront costs in some cases, can be realistically implemented in health systems, as these initial investments may be amortized over time while yielding favorable outcomes [18,40]. In this respect, the study provides clear support for considering immersive training not as a remote or impractical option, but as a feasible and potentially valuable strategy for improving home care safety [25].

### Practical Implications

These findings have direct implications for the real-world implementation of caregiver training. Our direct cost per participant for 360VT is consistent with non–head-mounted deployments (tablets and smartphones) using reusable content, and far below immersive configurations based on Microsoft HoloLens (higher entry cost) [34]. This helps explain why 360VT is particularly efficient in this setting. Accordingly, a three-tier strategy is proposed: (1) adopt 360VT as the standard to achieve wide coverage and rapid scale-up, combining outcome improvement with savings; (2) use VR selectively for high-risk tasks or caregivers with high burden, where the largest incremental effects can be achieved at an acceptable unit cost; and (3) add a psychoeducational module to extend low-cost error reduction when resources or time for in-person sessions are limited. This mix balances budget impact and effect size and is compatible with progressive rollout in public systems.

For public systems, the mixed strategy is especially reasonable; 360VT offers a low-cost, highly scalable solution for broad coverage and at-home reinforcement, while VR can be reserved for high-impact modules (eg, critical tasks or high-risk errors) in cohorts where greater clinical effect justifies the cost differential. The approach becomes even more favorable when material reuse is ensured (successive cohorts over ≥3 y), which the literature notes as key for VR to become cheaper than traditional simulation in the medium term.

### Strengths

This analysis draws on real-world, multisite data collected under a shared core protocol, enhancing external validity and comparability across settings. It adopts a societal perspective with explicit valuation of caregiver time in line with the consistency rule, ensuring a comprehensive accounting of resources. Costs and consequences are presented through a transparent CCA with disaggregated outcomes and clearly labeled ICERs, and robustness is examined via deterministic sensitivity analysis focused on downstream health care use as a key cost driver. Compared with the existing literature, this work adds novelty by integrating these elements in a single pragmatic evaluation—particularly the explicit treatment of caregiver time under a societal perspective, the use of a fully transparent CCA with labeled ICERs per outcome, and the targeted sensitivity analysis on downstream usage, which are seldom reported together in studies of simulation-based training.

### Limitations

Outcomes are self-reported, introducing risks of recall and social desirability bias and possible Hawthorne effects. In addition, allocation to different training modalities was not randomized; this is a study limitation, as residual selection cannot be excluded. The sample size may be insufficient for subgroup analyses (eg, by burden level, sex, or task type), and baseline imbalances (eg, ZBI scores) may persist despite adjustments; if adjustment is not feasible, residual confounding should be acknowledged. In addition, potential clustering by site could contribute unmodeled variance if not explicitly accounted for. The 3-month horizon may underestimate the durability of effects. On the costing side, indirect costs were not included, which could underestimate the total economic impact. Assumptions for downstream health care use—namely, a fixed composite unit cost and a base 2% trigger proportion—were explored in deterministic sensitivity analyses, yet real-world variation may differ by context and task. Transferability to other systems will require local costing, and base-year prices and inflation adjustments (deflators and wage structures) may differ across settings, limiting international comparability. Analytically, while a cost-consequence framework with labeled ICERs improves transparency, it can hinder comparability with classical cost-effectiveness or cost-utility evaluations, and ICER interpretation remains contingent on the stated assumptions, perspective, and short follow-up. Finally, external validity is restricted to the contexts and health systems from which the data were drawn, and the estimation of adverse event–related costs can vary widely with harm severity; the conservative valuation adopted here may underestimate costs in more severe scenarios. Finally, although CIs were estimated for all ICERs, some comparisons, particularly those involving small differences in effectiveness between interventions, produced relatively wide CIs. This indicates statistical uncertainty around certain incremental estimates and suggests that cost-effectiveness comparisons between active interventions should be interpreted with caution.

### Future Research

Patient safety in the home is a priority for health and care systems [1,2]. Future studies should use pragmatic designs that enable institutional scale-up (eg, cluster trials in primary care and home-care services with concurrent controls). Psychoeducational interventions should be reinforced with explicit content on double victimization—posterror support for informal caregivers to address feelings of responsibility for unwanted, avoidable harm—to reduce emotional burden when incidents have serious consequences; brief coping modules, disclosure guides, and peer-support referral can be tested for effects on burden and reattendances. Task-specific analyses are needed to determine which activities benefit most from 360VT versus VR and when a reinforced psychoeducation module suffices as a first-line option.

Longer follow-up is warranted to capture maintenance of effects, reuse of materials, and household spillovers, alongside objective measures via electronic health records and pharmacy data, and service-use end points (reattendances and emergency visits). Studies should establish willingness-to-pay thresholds (eg, per ZBI-7 point reduced) and evaluate stepped models of care (360VT as the base, VR for higher-risk tasks, and psychoeducation reinforced after incidents). Implementation and equity evaluations should address uptake, usability, digital literacy, and gender differences. Additional priorities include mediation or moderation analyses of the bidirectional burden-error relationship, assessment of transferable competencies to formal caregivers in the public system, and sustainability analyses (amortization, maintenance, and innovative public procurement) to inform area-level decision-making.

### Conclusions

This study is innovative in providing a real-world comparative CCA of multiple training modalities for informal caregivers under a shared protocol and from a societal perspective. Unlike most existing studies, which focus on feasibility, acceptability, or single modalities, it compares immersive and nonimmersive approaches using harmonized safety, caregiver-burden, and downstream cost outcomes. It brings comparative economic evidence to a field where such analyses remain limited and have clear real-world implications. Technology-enhanced training for informal caregivers reduces caregiving errors at home at a very low unit cost, thereby supporting improvements in home-care safety. A stepped pathway, 360VT for scalable coverage, VR for targeted reinforcement, and psychoeducation as a low-cost complement, offer a pragmatic alignment of effect size and budget impact for real-world programs and warrant more detailed evaluation in future studies.

## Supplementary material

10.2196/85141 Multimedia Appendix 1 Caregiving tasks organized by thematic blocks.

10.2196/85141 Multimedia Appendix 2 Psychoeducational intervention.

10.2196/85141 Checklist 1 CHEERS checklist.

## Abbreviations

360VT: 360° video training
AR: augmented reality
CCA: cost-consequence analysis
CHEERS: Consolidated Health Economic Evaluation Reporting Standards
ICER: incremental cost-effectiveness ratio
VR: virtual reality
XR: extended reality
ZBI-7: 7-item Zarit scale
