# Supplementary material for: Improving Home Care Safety Among Informal Caregivers Through Immersive Digital Simulation: Secondary Analysis of 3 Coordinated Intervention Studies
Source: J Med Internet Res. 2026 Jul 9;28:e85141. doi: 10.2196/85141 (PMC13349226; doi:10.2196/85141)
Supplement: Multimedia Appendix 1 [file jmir-v28-e85141-s001.docx]

**Oncology patient pack**

Hand hygiene

House of horrors

Daily hygiene and correct selection of elements in a hygiene process

Diaper change

Administration of subcutaneous drugs: insulin, glucagon, heparin

Administration of subcutaneous drugs: morphine and derivatives

Port-a-Cath

**Heart failure patient pack**

Hand hygiene

House of horrors

Medication errors in tablets, sachets, capsules, syrups, and oral solutions

Daily hygiene and correct selection of elements in a hygiene process

Cardiopulmonary resuscitation

Patient with heart failure: weight changes, diet adjustments

Blood pressure monitoring

**Basic hygiene pack**

Hand hygiene

House of horrors

Medication errors in tablets, sachets, capsules, syrups, and oral solutions

Daily hygiene and correct selection of elements in a hygiene process

Diaper change

Patient transfers to armchair, bathroom, or shower from bed or wheelchair

Prevention of pressure ulcers

Administration of subcutaneous drugs: insulin, glucagon, heparin

Prevention of bronchial aspiration in patients with dysphagia

Blood pressure monitoring

**Mobility/rehabilitation pack**

Hand hygiene

House of horrors

Medication errors in tablets, sachets, capsules, syrups, and oral solutions

Diaper change

Patient transfers to armchair, bathroom, or shower from bed or wheelchair

Administration of subcutaneous drugs: insulin, glucagon, heparin

Prevention of bronchial aspiration in patients with dysphagia

Patient with orthosis

Patient requiring walking rehabilitation following a hip fracture

Care for the caregiver

**Post-stroke pack**

House of horrors

Patient transfers to armchair, bathroom, or shower from bed or wheelchair

Prevention of pressure ulcers

Administration of subcutaneous drugs: insulin, glucagon, heparin

Prevention of bronchial aspiration in patients with dysphagia

Patient requiring speech therapy for improvements in speech and swallowing
