# Supplementary material for: Improving Home Care Safety Among Informal Caregivers Through Immersive Digital Simulation: Secondary Analysis of 3 Coordinated Intervention Studies
Source: J Med Internet Res. 2026 Jul 9;28:e85141. doi: 10.2196/85141 (PMC13349226; doi:10.2196/85141)
Supplement: Multimedia Appendix 2 [file jmir-v28-e85141-s002.docx]

**Objective:** To achieve safer medication use and caregiving practices at home

**Session duration:** 1 hour 30 minutes

**Participant profile:** 10-12 informal caregivers

**Contents:**

Session 1: Introduction to safe care, medication management at home, information resolution, and effective communication

- Introduction of moderators and group norms
- Introduction of group members (profile of the person they care for, time dedicated to caregiving)
- Overview of sessions structure
- Presentation of the intervention objectives

Open-ended question 1: *What is your typical day like as a caregiver?*

- Explanation of medication errors

Open-ended question 2: *What difficulties or barriers do you face in your daily caregiving related to medication and care?*

- Most common errors and critical moments

Open-ended question 3: *Where do you store medications at home?*

- Information on medication packaging
- Recommendations for proper medication storage

Open-ended question 4: *How do you organize medication at home for the person you care for?*

- Strategies to support medication adherence

Open-ended question 5: *What strategies do you use to resolve doubts about medication and health?*

- Preparing for medical appointments
- Consulting reliable websites

Open-ended question 6: *Who is involved in the care process?*

- Importance of communication with the patient, family members, and other caregivers
- Communicating errors

Session 2: Practical workshop

- Blood pressure measurement
- CPR (Cardiopulmonary Resuscitation)
- Self-care
